# Study of Chemical Enhancement Mechanism in Non-plasmonic Surface Enhanced Raman Spectroscopy (SERS)

**DOI:** 10.3389/fchem.2019.00582

**Published:** 2019-08-20

**Authors:** Jayeong Kim, Yujin Jang, Nam-Jung Kim, Heehun Kim, Gyu-Chul Yi, Yukyung Shin, Myung Hwa Kim, Seokhyun Yoon

**Affiliations:** ^1^Department of Physics, Ewha Womans University, Seoul, South Korea; ^2^Department of Physics and Astronomy, Institute of Applied Physics, Research Institute of Advanced Materials, Seoul National University, Seoul, South Korea; ^3^Department of Chemistry and Nanoscience, Ewha Womans University, Seoul, South Korea

**Keywords:** surface enhanced Raman scattering, chemical enhancement, enhancement mechanism, charge transfer, semiconductor microstructure

## Abstract

Surface enhanced Raman spectroscopy (SERS) has been intensively investigated during the past decades for its enormous electromagnetic field enhancement near the nanoscale metallic surfaces. Chemical enhancement of SERS, however, remains rather elusive despite intensive research efforts, mainly due to the relatively complex enhancing factors and inconsistent experimental results. To study details of chemical enhancement mechanism, we prepared various low dimensional semiconductor substrates such as ZnO and GaN that were fabricated via metal organic chemical vapor deposition process. We used three kinds of molecules (4-MPY, 4-MBA, 4-ATP) as analytes to measure SERS spectra under non-plasmonic conditions to understand charge transfer mechanisms between a substrate and analyte molecules leading to chemical enhancement. We observed that there is a preferential route for charge transfer responsible for chemical enhancement, that is, there exists a dominant enhancement process in non-plasmonic SERS. To further confirm our idea of charge transfer mechanism, we used a combination of 2-dimensional transition metal dichalcogenide substrates and analyte molecules. We also observed significant enhancement of Raman signal from molecules adsorbed on 2-dimensional transition metal dichalcogenide surface that is completely consistent with our previous results. We also discuss crucial factors for increasing enhancement factors for chemical enhancement without involving plasmonic resonance.

## Introduction

Since its first observation and following explanation by pioneers (Fleischmann et al., 1974; Jeanmaire and Van Duyne, 1977; Moskovits, 1985; Otto, 1991; Smith and Dent, 2005; Stiles et al., 2008; Schlücker, 2014), surface enhanced Raman spectroscopy (SERS) has been a subject of intense research in various disciplines, especially in analytical chemistry utilizing its very high sensitivity that might overcome main disadvantage of the signal weakness of Raman scattering spectroscopy (Chan et al., 2003; Haynes and Van Duyne, 2003; Tian and Ren, 2004; Haynes et al., 2005; Zhang et al., 2005, 2008; Le Ru et al., 2008; Wang et al., 2010). It is reported that SERS enhancement factor (EF) can be as high as 10^12^ in some cases (David et al., 2010) indicating that unprecedented area of analyses such as Raman fingerprinting of a very small amount of materials, even of a single molecule may be possible, and hence enormous research efforts have been exerted over the decades (Kneipp et al., 1997; Nie and Emory, 1997; Stiles et al., 2008). Recent applications of SERS include, for example, employing various nanostructured materials (Wang Y. et al., 2012; Jin, 2013; Li et al., 2013, 2017; Xu et al., 2013; Lin et al., 2015; Bakan et al., 2016; Wang et al., 2016; Chowdhury et al., 2018; Yu et al., 2019). SERS is also very interesting because it inherently involves light-matter interaction in small scale where quantum mechanical effects might be crucial. In this regard, SERS attracts attention from research communities that emphasize basic research and that are rather application-oriented as well.

By now, it is well-accepted that the largest contribution for the EF is coming from so-called electromagnetic mechanism. In the electromagnetic mechanism, it is explained that the Raman signal enhancement occurs as a result of surface plasmonic resonance, i.e., when the energy of the laser excitation is close to the surface plasmon energy of a substrate which is often made of noble metal, Raman response is strongly enhanced. Obviously there should be another mechanism to explain SERS results that depend not only on substrates but also analyte molecules, which is referred to as chemical mechanism (Wang X. et al., 2012; Alessandri and Lombardi, 2016). As a matter of fact, chemical enhancement (CE) is rather inclusive nomenclature than a specific phenomenon that comes from a definite origin. For example, CE includes several different transitions/processes. In case of a metal substrate, the charge transfer transition between highest occupied molecular orbital (HOMO) of analyte molecules and the fermi level of metal is most relevant. For dielectric substrates, charge transfer transition between either HOMO of molecules and the conduction band (CB) edge of substrate material or valence band (VB) of substrate material and lowest unoccupied molecular orbital (LUMO) of molecules is crucial. Moreover, electronic transition from HOMO to LUMO or transition between VB and CB of substrate material can also contribute to the enhancement of Raman signal by resonance processes that lead to surface enhanced resonance Raman spectroscopy (SERRS). All of the above effects can contribute to enhancement of Raman response and tracking the exact origin of a particular enhancement is not always trivial. Even though there is some guidance from theoretical side (Lombardi and Birke, 2007, 2012, 2014), at this point, if possible, experimental ways to single out the main effect for CE is needed. With this information, we can have better understanding of chemical enhancement mechanism and we can establish microscopic theory of CE.

In this study, we used compound III-V semiconductor (ZnO and GaN) microstructure and 2-dimensional transition metal dichalcogenide (WS_2_) as substrates to study charge transfer (CT) transitions and their influence on enhanced Raman spectra. By using semiconductor materials as substrates, we could safely exclude surface plasmon resonance as the origin of Raman enhancement in our measurements. Another advantage of using semiconductor materials is that we can fully use current state-of-art semiconductor technology for preparing substrates. For example, ZnO and GaN microstructures we used can be made rather easily by following well-defined procedures resulting good reproducibility and the manufacturing cost is not high (Kim et al., 2012; Park et al., 2015). It is well-known that ZnO (Zhao et al., 2012), GaN (Jewett et al., 2012), and WS_2_ (Goldman et al., 2015) are biocompatible so together with easy and highly reproducible fabrication make semiconducting materials as quite promising SERS substrates with wide applicability. We observed clear SERS effects from all combinations of our samples but there is indication that the enhancement is asymmetric, that is, there might be a preferential route of charge transfer between substrates and analytes for exhibiting large EF. We suggest that there can be a dominant enhancement mechanism in CE that can lead us to design combinations of materials showing maximal Raman enhancement effect.

## Experimental

### Fabrication of SERS Substrate Samples

The position-controlled ZnO microwalls are grown on chemical vapor deposition (CVD) graphene films through the catalyst-free CVD process. We obtained ZnO microwall growth selectivity by depositing a silicon dioxide (SiO_2_) growth mask on the graphene films and patterning hole array on the growth mask layer using E-beam lithography and etching techniques. Dimensional parameters including the heights and diameters of microwalls can be controlled by modifying the lithographic pattern mask or varying the growth parameters of catalyst-free CVD process. In this work, highly oriented and ordered ZnO mircrowalls with an outer diameter of 4 μm and a height of 4 μm were typically employed. For GaN substrates, GaN layers were over-grown laterally from pre-fabricated ZnO microstructures using a similar MOVPE process with tri-methyl gallium and ammonia as sources. The detailed process for growing ZnO/GaN microstructure is described in previous reports (Kim et al., 2012, 2019; Park et al., 2015).

The vertically aligned single crystalline tungsten disulfide WS_2_ nanosheets was synthesized via the chemical vapor deposition (CVD) method on a Si wafer. The process to form the WS_2_ were as follows. First of all, the quartz boat with tungsten hexachloride (WCl_6_, Sigma-Aldrich) was placed in the center of the quartz tube and another quartz boat with sulfur powder (Sigma-Aldrich, 99.98%) was held 14.5 cm distant from it. The Si substrate was placed face-down above the quartz boat with tungsten hexachloride. Before the chemical reaction, flushing the quartz tube by carrier gas helium (He, 99.999%) for 10 min by 400 sccm (standard cubic centimeter per minute) to remove impure gases in quartz tube. When the flushing was finished, the furnace was heated up to 550 through the speed rate of 26 per minute condition and kept the temperature at 550°C for 20 min. The carrier gas, helium (He) was flowed by 10 sccm for the whole process since heating up the furnace. After finishing the chemical reaction, stop flowing carrier gas was off at the same time. The furnace was turned off after chemical reaction and cooled down gradually until to room temperature.

### Preparation for SERS Measurement Samples—Molecule Deposition

We prepared SERS samples by two methods of immersing and drop casting. For ZnO substrates, we immersed the substrates in molecular solution with 10^−4^M concentration for 2 h, and washed with deionized water. We used three different molecules (4-MPY, 4-MBA, 4-ATP) for the molecular solution for each ZnO substrates. For GaN substrates, we deposited the molecules (4-MPY, 4-MBA, 4-ATP) by drop casting as follows. 10 μL of 10^−3^ M 4-Mpy solution was prepared and was divided into 4 equal droppings of 2.5 μLs each that was dropped on a substrate and was left dried naturally. We also using drop casting method for depositing R6G molecules on WS_2_ nanoflower structure.

### SERS Measurements

Room temperature Raman scattering spectra of ZnO and GaN microstructure samples with 3 different molecules (4-MPY, 4-MBA, 4-ATP) adsorbed and R6G adsorbed WS_2_ samples were measured by using a McPherson 207 spectrometer equipped with a nitrogen-cooled charge-coupled-device (CCD) array detector. The ZnO microstructure samples were excited with 532.0 nm (2.33 eV) DPSS (diode-pumped solid state) laser, focused to ~1 μm diameter spot using a microscope objective (x50). The GaN microstructure samples were excited with 514.5 nm (2.41 eV) Ar+ ion laser, also focused to ~1 μm diameter spot using a microscope objective (x50). The WS_2_ samples were excited with three different excitation wavelengths, 488.0 nm (2.54 eV), 532.0 nm, 632.8 nm (1.96 eV). The excitation power was kept <0.2 mW to avoid laser heating.

## Results and Discussions

**Figure 1:**
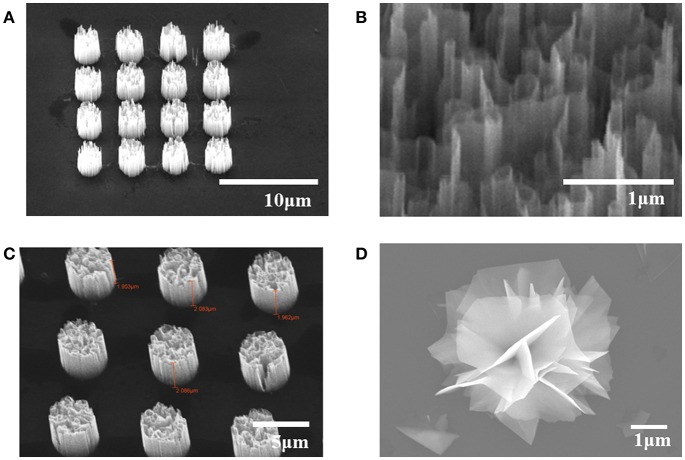
SEM images of **(A)** GaN microrod array **(B)** nanowall structure in a microrod **(C)** ZnO microrod array **(D)** CVD grown WS_2_ nanoflower.

Figure 1 shows scanning electron microscope (SEM) images of samples we used. Figure 1A is a picture of highly oriented GaN microrod array. Figure 1B shows the maze-like structure inside each GaN microrods, which provides large surface area. Figure 1C is highly oriented ZnO microrod array and Figure 1D shows a WS_2_ “nanoflower” flake structure. The samples we used exhibit high crystalline quality with little defects and display excellent optical characteristics, as shown in previous reports (Kim et al., 2012, 2017, 2019; Park et al., 2015).

**Figure 2:**
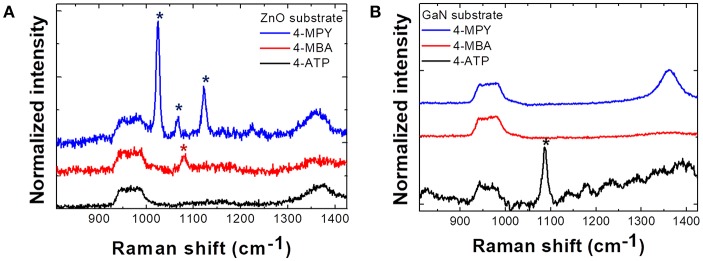
Raman spectra of three different molecules (4-MPY, 4-MBA, 4-ATP) adsorbed on **(A)** ZnO microrod array substrate and **(B)** GaN microrod array substrate. Phonon intensity in each spectrum is normalized to Si phonon (520 cm^−1^) intensity. Raman peaks of 4-MPY and 4-MBA molecules are enhanced only on the ZnO substrate and Raman peak of 4-ATP molecules is enhanced only on the GaN substrate. ^*^Indicates the peak positions of each molecules (4-MPY, 4-MBA, 4-ATP).

Figure 2 illustrates Raman spectra from (a) ZnO and (b) GaN semiconductor microrod arrays. From the spectra it is clear that the enhancement of Raman signal is quite selective with respect to the combinations of adsorbed molecules and substrates. In ZnO substrates, only 4-MPY shows clearly enhanced response and signal from 4-ATP is enhanced only on GaN substrates. This can be explained by CE, that is, the Raman signal of analyte molecules is strongly enhanced when the laser excitation energy matches with the CT transition energy between substrates and analyte molecules. This point will be discussed further with explanations of **Figure 4**.

**Figure 3:**
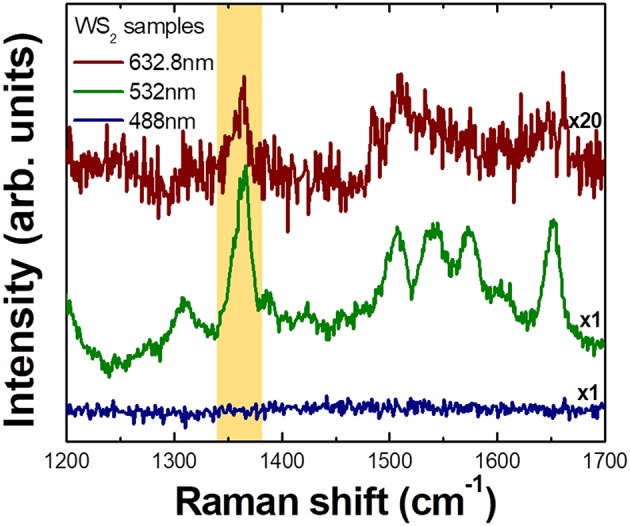
Wavelength dependent Raman spectra of R6G molecules adsorbed on CVD grown WS_2_ nanoflower substrate measured with 3 different excitation lasers. Phonon intensity in each spectrum is normalized to Si phonon (520 cm^−1^) intensity. The largest enhancement is observed at 532.0 nm excitation and significantly weaker but observable enhancement is observed at 632.8 nm excitation. No observable enhancement is seen under 488.0 nm illumination.

In Figure 3, excitation wavelength dependence of Raman spectra of R6G molecules adsorbed on CVD grown WS_2_ nanoflower substrates is shown. It is clear that signal is strongly enhanced when the excitation energy is 2.33 eV (532.0 nm), is significantly weaker but still observable for 1.96 eV (632.8 nm) excitation, and is not observable under 2.54 eV (488.0 nm) illumination. As a matter of fact, there are numerous works reporting that there is a SERS like effect from TMDC materials (Sun et al., 2014; Lee et al., 2016; Muehlethaler et al., 2016; Miao et al., 2018; Zheng et al., 2018). Our observation can again be explained by CE that is related with light induced charge transfer transition between substrates and analytes. Thus, the selective Raman enhancement of analyte molecules is not just a characteristic for III–V semiconductor microstructures but also is observed in two-dimensional transition metal dichalcogenide (TMDC) materials suggesting that it is rather universal phenomenon in molecule-semiconductor system.

**Figure 4:**
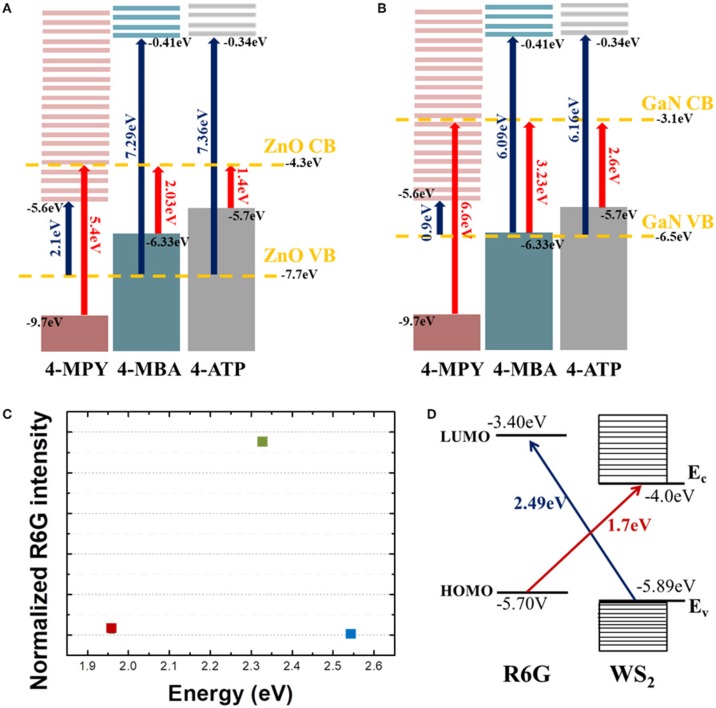
Energy level diagrams of three molecules (4-MPY, 4-MBA, 4-ATP) with **(A)** ZnO and **(B)** GaN. Bands of each semiconductor are denoted by yellow dashed lines. All vertical arrows indicate possible charge transfer routes between molecules and semiconductor substrates. Blue arrows denote charge transfer transition from semiconductor valence band (VB) to LUMO of molecules, and red arrows denote charge transfer transition from HOMO of molecules to semiconductor conduction band (CB). **(C)** Normalized (to Si phonon intensity) intensity of R6G 1368 cm^−1^ mode with three different excitation laser wavelengths. **(D)** Energy level schematics of R6G and WS_2_. The red arrow denotes a charge transfer route from HOMO of R6G to WS_2_ CB, and the blue arrow denotes a charge transfer route from WS_2_ VB to LUMO of R6G.

Energy band schematics and relevant electronic energy diagrams are shown in Figure 4. For CE, there are four energy scales that need to be considered. The first is the molecular transition from HOMO to LUMO, related with “A-term” in Herzberg-Teller (HT) treatment (Albrecht, 1961; Schatz and Ratner, 2002), the second is a charge transfer transition from molecular HOMO to CB of semiconductor substrates (B-term in HT picture), the third is a charge transfer transition from the VB of semiconductor substrates to molecular LUMO (HT's C-term), and the fourth is the band or excitonic transition from VB to CB of semiconductor substrates. From our previous work, we can safely exclude the HOMO to LUMO transition and the VB to CB transition as main cause for Raman enhancement (Kim et al., 2019). Our results can rather be explained by transitions described by either HT's B- or C-term and resulting enhancement. In Lombardi and Birke's pioneering work they explained the chemical enhancement mechanism of SERS by resonance process of which the relevant energy is the difference between either HOMO and CB (B-term) or VB to LUMO (C-term) where light induced charge transfer transition can occur (Lombardi and Birke, 2014). In their theory, however, there is no a priori difference between B- and C-terms, that is, large enhancement can occur due to any of the two processes and there would be no preference or advantage from one process over another. There can be even more pathways for charge transfer such as transition involving the surface state of semiconductor, charge transfer complex, and/or excitonic states, for example (Kneipp et al., 2018). In any of those current approaches, there is no dominant process over other ones.

Recently, we reported that there might be a preferential route for CE by observing selective enhancement that strongly depends on the relative positions of each energy level (Kim et al., 2019). Interestingly, the EF calculated for a transition due to C-term (transition from VB to LUMO) was found to be at least 100 times larger than that for a transition due to B-term (transition from HOMO to CB) suggesting that utilizing a charge transfer from semiconductor substrates to molecular LUMO would be more efficient way to attain large EF in chemical enhancement. From Figure 4A, we can see that how charges are transferred for each molecule. When energy excitation energy of 2.33 eV is applied to ZnO and molecules, charge transfer transition can only be caused by C-term for 4-MPY (denoted by blue arrow) and B-term for 4-MBA (denoted by red arrow), respectively. The other way, red arrow (B-term) for 4-MPY and blue arrow (C-term) for 4-MBA, of transition cannot occur with 2.33 eV excitation. If we compare Raman intensities associated with charge transfer, C-term related one (4-MPY) is much larger than B-term related one (4-MBA). Similarly, charge transfer between GaN substrate and molecules can only occur through B-term for 4-ATP (Figure 4B) with 2.41 eV excitation. This agrees well with our experimental results shown in Figures 2A,B. This observation of asymmetric enhancement is also evident from the R6G on WS_2_ as shown in Figures 4C,D. Figure 4C shows the normalized R6G intensity to Si phonon intensity as a function of excitation energy. Comparing this with the energy band diagram of R6G and WS_2_ in Figure 4D, we can see that large enhancement at 2.33 eV observed in Figure 3 is associated with C-term (blue arrow in Figure 4D). The much weaker enhancement at 1.96 eV observed in Figure 3 is due to B-term (red arrow in Figure 4D).

Our current observations from three different material systems are completely consistent with our previous reports (Shin et al., 2014; Kim et al., 2019). That is, chemical enhancement seems to occur asymmetrically. Stronger enhancement with larger EF is associated with charge transfer transition from semiconductor substrates to analyte molecules. More work need to be done before we completely understand why there seems to be a dominant route showing a larger EF for CE, but possible cause would be associated with density of states, existence of excitonic levels, coordination and/or orientation of molecules on the surface of substrates, for example. Since CE is caused by “borrowing intensity” from transitions of various origins, it is not always easy to single out which one is the most relevant or irrelevant to Raman enhancement. From our measurements, we could suggest that the most important transition for attaining larger EF is C-term related one. In other words, the dominant process for chemical SERS effect is associated with charge transfer transition from semiconductor VB to molecular LUMO.

## Conclusions

We measured Raman response from adsorbed molecules on III–V semiconductor microstructures and thin TMDC materials. In non-plasmonic condition where the energy of excitation laser is far from that of surface plasmon of substrates SERS is thought to be associated with charge transfer transitions, that is referred to as chemical enhancement. In theoretical prediction, there is no preferential route or dominant channel for chemical enhancement, however, we found that the charge transfer transition from semiconductor substrates to molecular LUMO may play a role as an efficient or a dominant process for attaining a large EF. We observed the above results in two independent systems, one in III–V compound semiconductor microstructure and the other in thin TMDC materials. Our finding can provide important information for understanding chemical SERS effects microscopically.

## Data Availability

All datasets generated for this study are included in the manuscript and/or the supplementary files.

## Author Contributions

JK carried out most of the experimental work and analyses and drafted the paper. YJ helped with the experimental work and analyses. N-JK contributed initial design of the study. HK and G-CY wrote sections of the manuscript and provided the III–V semiconductor microrod array samples. YS and MK wrote sections of the manuscript and provided the TMDC samples. SY designed the project and wrote the paper. All authors have approved the final version of the manuscript.

### Conflict of Interest Statement

The authors declare that the research was conducted in the absence of any commercial or financial relationships that could be construed as a potential conflict of interest.
